# Supplementary figures and images for: Integrated Transcriptome and Untargeted Metabolomic Analyses Revealed the Role of Methyltransferase Lae1 in the Regulation of Phospholipid Metabolism in Trichoderma atroviride
Source: J Fungi (Basel). 2023 Jan 14;9(1):120. doi: 10.3390/jof9010120 (PMC9864869; doi:10.3390/jof9010120)

Fig S1.

A.

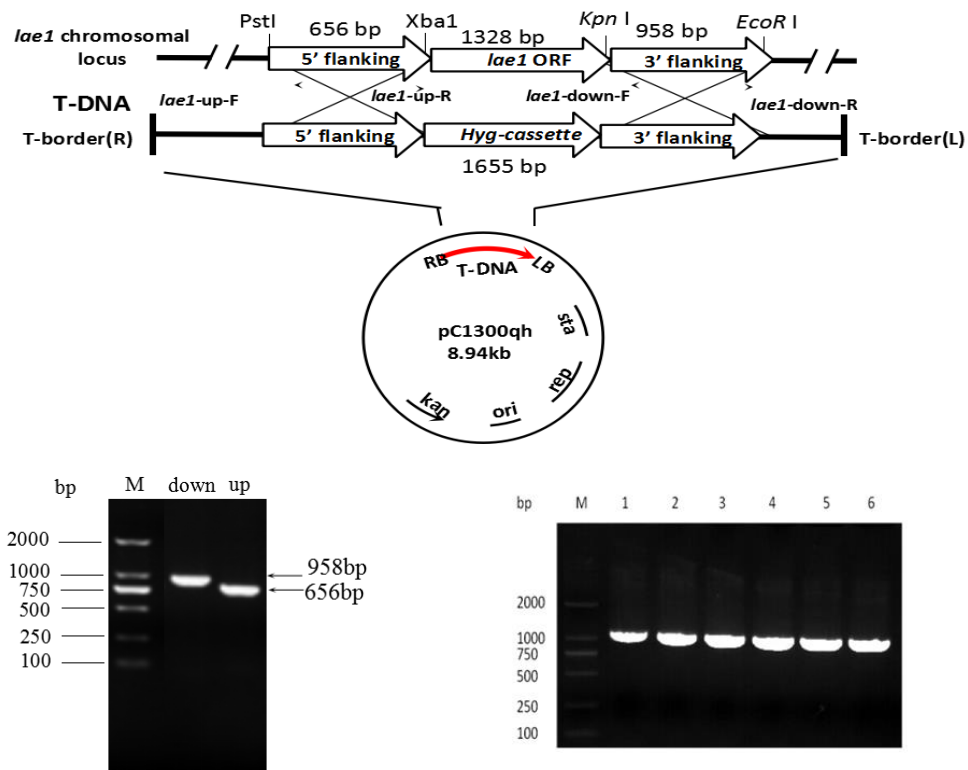

B.

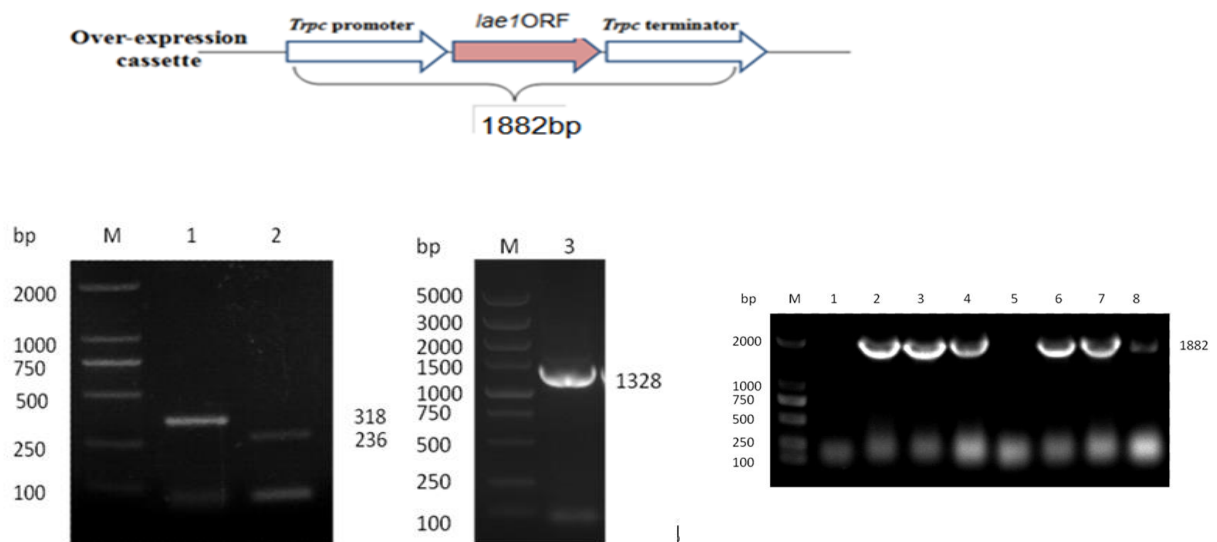

Supplement: Supplementary file 1 [file jof-09-00120-s001.zip › Figure S1.pdf]

**A**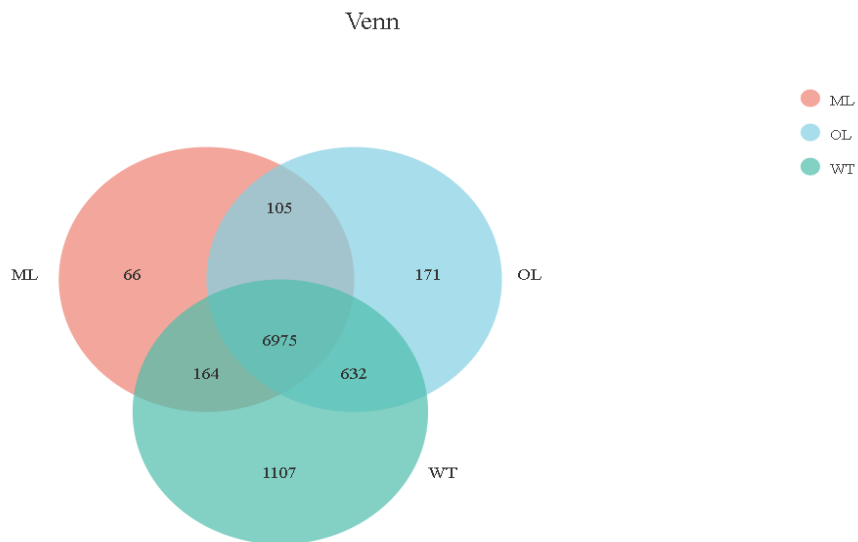**B**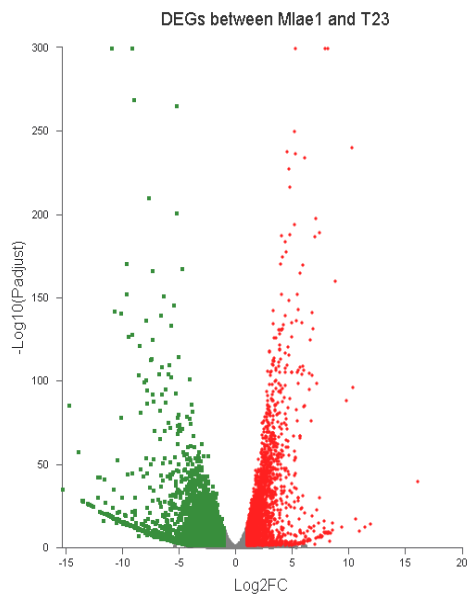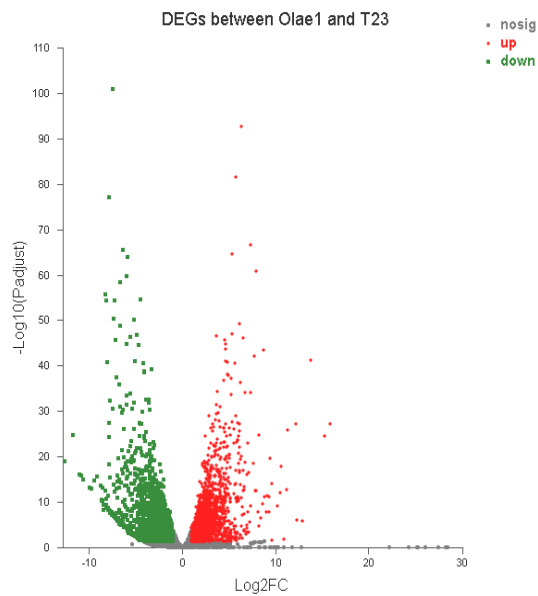

Supplement: Supplementary file 1 [file jof-09-00120-s001.zip › Figure S2.pdf]

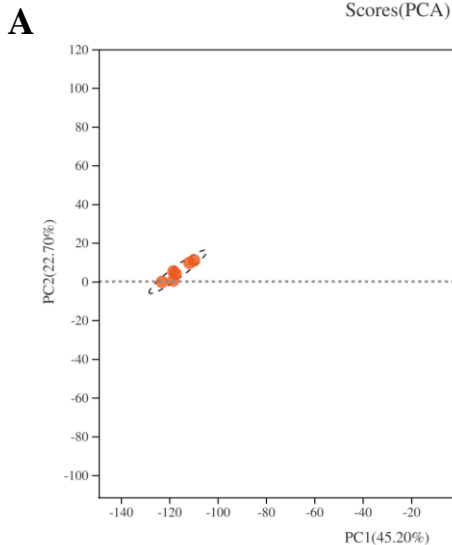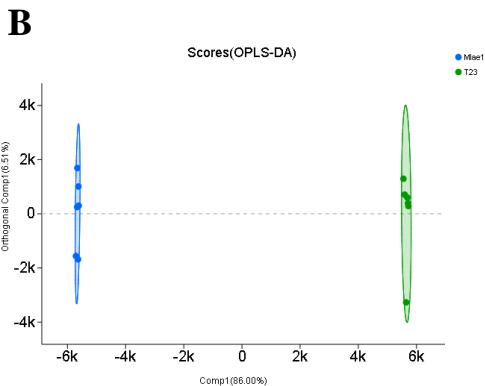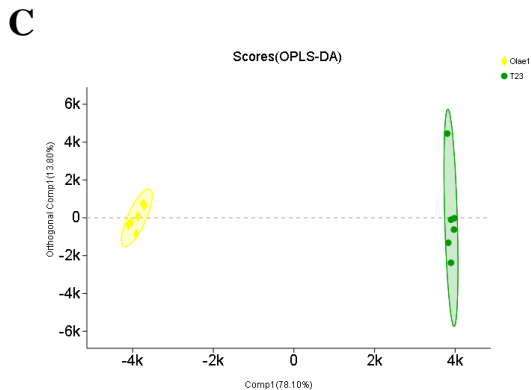

Supplement: Supplementary file 1 [file jof-09-00120-s001.zip › Figure S3.pdf]

# KEGG Enrichment Analysis(total)

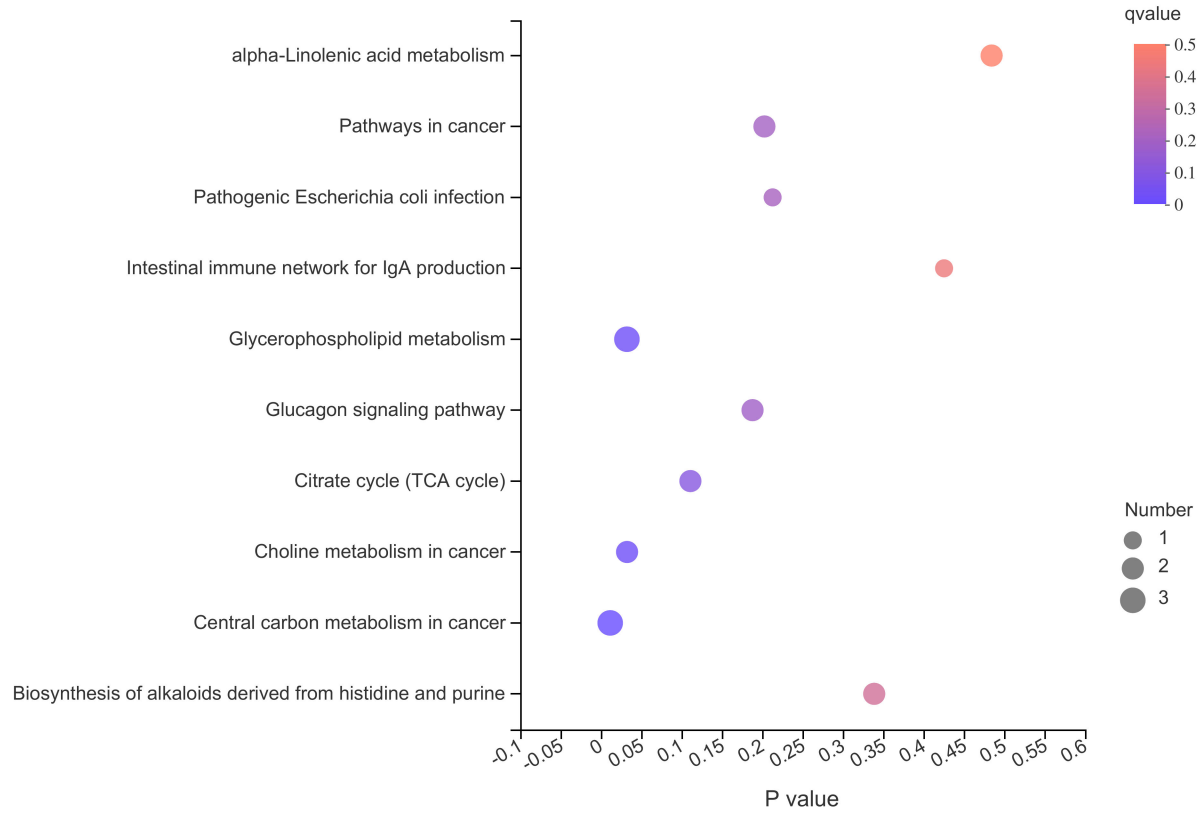

Supplement: Supplementary file 1 [file jof-09-00120-s001.zip › Figure S4.pdf]
